# Use of and access to health services in Brazil, 2013 National Health Survey

**DOI:** 10.1590/S1518-8787.2017051000074

**Published:** 2017-06-01

**Authors:** Sheila Rizzato Stopa, Deborah Carvalho Malta, Camila Nascimento Monteiro, Célia Landmann Szwarcwald, Moisés Goldbaum, Chester Luiz Galvão Cesar

**Affiliations:** I Programa de Pós-Graduação em Saúde Pública. Faculdade de Saúde Pública. Universidade de São Paulo. São Paulo, SP, Brasil; II Departamento de Enfermagem Materno Infantil e Saúde Pública. Escola de Enfermagem. Universidade Federal de Minas Gerais. Belo Horizonte, MG, Brasil; III Núcleo de Indicadores e Sistemas de Informação. Hospital Israelita Albert Einstein. São Paulo, SP, Brasil; IV Instituto de Comunicação e Informação Científica e Tecnológica em Saúde. Fundação Oswaldo Cruz. Rio de Janeiro, RJ, Brasil; V Departamento de Medicina Preventiva. Faculdade de Medicina. Universidade de São Paulo. São Paulo, SP, Brasil; VI Departamento de Epidemiologia. Faculdade de Saúde Pública. Universidade de São Paulo. São Paulo, SP, Brasil

**Keywords:** Health Services, supply & distribution, Health Services Accessibility, Equity in Access, Socioeconomic Factors, Health Surveys, Serviços de Saúde, provisão & distribuição, Acesso aos Serviços de Saúde, Equidade no Acesso, Fatores Socioeconômicos, Inquéritos Epidemiológicos

## Abstract

**OBJECTIVE:**

To analyze the use of health services in the Brazilian population by sociodemographic factors, according to data from the 2013 Brazilian National Health Survey.

**METHODS:**

The study analyzed data from 205,000 Brazilian citizens in all age groups who participated in the Brazilian National Health Survey, a cross-sectional study carried out in 2013. Prevalence and confidence intervals were estimated for indicators related to access to and use of health services according to age group, level of education of head of household, and Brazilian macroregions.

**RESULTS:**

Among individuals who sought health services in the two weeks prior to the survey, 95.3% (95%CI 94.9–95.8) received care in their first visit. Percentages were higher in the following groups: 60 years of age and over; head of household with complete tertiary education; living in the South and Southeast regions. In addition, 82.5% (95%CI 81.2–83.7) of individuals who received health care and prescriptions were able to obtain all the necessary medicines, 1/3 of them from SUS. Less than half the Brazilian population (44.4%; 95%CI 43.8–45.1) visited a dentist in the 12 months prior to the survey, with smaller percentages among the following groups: 60 years of age or older; head of household with no education or up to incomplete elementary; living in the North region of Brazil.

**CONCLUSIONS:**

People living in the South and Southeast regions still have greater access to health services, as do those whose head of household has a higher level of education. The (re)formulation of health policies to reduce disparities should consider differences encountered between regions and social levels.

## RESUMO

**OBJETIVO:**

Descrever o uso de serviços de saúde na população brasileira segundo fatores sociodemográficos, de acordo com dados da Pesquisa Nacional de Saúde, 2013.

**MÉTODOS:**

Foram analisados dados referentes a 205 mil brasileiros, de todas as faixas etárias, que participaram da Pesquisa Nacional de Saúde, estudo transversal conduzido em 2013. Calcularam-se as prevalências e seus intervalos de confiança para indicadores referentes ao acesso e a utilização dos serviços de saúde, segundo grupos de idade, nível de instrução do chefe da família e macrorregiões do país.

**RESULTADOS:**

Dentre os indivíduos que procuraram o serviço de saúde nas duas semanas prévias à pesquisa, 95,3% (IC95% 94,9–95,8) conseguiu usá-lo na primeira vez que procurou. As proporções foram maiores: no grupo de 60 anos ou mais; cujo chefe da família tinha nível superior completo; e nas regiões Sul e Sudeste. Ainda, dos indivíduos atendidos e que tiveram medicamentos receitados, 82,5% (IC95% 81,2–83,7) conseguiram obter todos os medicamentos, sendo 1/3 pelo SUS. Menos da metade da população brasileira (44,4%; IC95% 43,8–45,1) consultou um dentista nos 12 meses anteriores à pesquisa, com proporções menores entre: indivíduos com 60 anos ou mais; cujo chefe da família não possuía nível de instrução ou tinha até o fundamental incompleto; e indivíduos que residiam na região Norte do país.

**CONCLUSÕES:**

Pessoas que residem nas regiões Sul e Sudeste ainda possuem maior acesso aos serviços de saúde, bem como aquelas cujo chefe da família tem maior nível de instrução. A (re)formulação de políticas de saúde no intuito de reduzir disparidades deve considerar as diferenças regionais e entre níveis sociais encontradas.

## INTRODUCTION

The use of health services results from several interacting factors, such as how users perceive them and their need and to what extent they are available^1,2^. Various conditions are associated with the offer of such services. Geographical accessibility and sociocultural and economic factors, for example, play an essential role in the demand for health services.^3,4^

The use of health services is determined by a need perceived by users based on their health situation and previous knowledge of the disease or condition, which in turn is influenced by sociodemographic factors^2,3^.

Access to health services also reflects existing inequalities and vulnerabilities among society, produced within the social framework. It is the state’s duty to ensure the principle of equity of the Brazilian Unified Health System (SUS), guaranteed by the Constitution, to offset the inequalities produced at social level^3,4^.

Population-based studies that address the use of health services are key to describing frequencies and trends. The findings of such studies lead to knowledge of access to and use of services in a given population, providing support for the planning and (re)formulation and management of health policies^2,5^.

Since the 1980s, the Brazilian National Household Survey Sample (PNAD) has been surveying the use of health services in its health supplements. However, these supplements have only been published regularly every five years since 1998. They have addressed access to and use of health services, self-reported morbidity, health plan coverage, health costs, and lifestyle^5^.

One of the objectives of the 2013 Brazilian National Health Survey (PNS) was to continue the work of the PNAD health supplements, but addressing a wider range of topics. PNS is a component of the Integrated Household Surveys System (SIPD) and is independent of the continuous PNAD, that is, it has its own design and was conceived to collect health information^6^. The second volume of PNS data allowed the monitoring of indicators of access to and use of health service addressed in previous PNAD supplements.^a^

This study aimed to analyze the use of health services in the Brazilian population by sociodemographic factors, according to data from the 2013 PNS.

## METHODS

This study analyzed data from the 2013 PNS, a population-based household survey conducted in Brazil by the Brazilian Institute of Geography and Statistics (IBGE), in partnership with the Brazilian Ministry of Health.

Cluster sampling was used in three selection stages. In the first stage, we stratified the primary sampling units composed of census tracts, using simple random selection. In the second stage, we randomly selected 10 to 14 households in each primary sampling unit. In the third stage, an adult was selected with equiprobability among all adults resident in the household. The census tracts used in the PNS were drawn from the IBGE SIPD, using the Master Sample of that system, with a wider geographical scope and gain in estimate precision^a^.

The estimated PNS sample size was approximately 81,000 households, with 81,254 households selected to compose the sample. Of these, 69,994 were occupied and home interviews were carried out in 64,348 of them. A total of 60,202 individual interviews was performed with selected adults in the households. The loss rate was 20.8% for home interviews and 25.9% for individual interviews. The PNS sample was planned considering the precision level desired for some estimates of specific indicators of interest. This strategy allowed the estimation of parameters at different geographical levels: federative units, capitals, metropolitan areas and the remaining federative units^a^.

Sample weights were defined for the primary sampling units, households and all residents, as well as for the selected resident. The latter was estimated considering the weight of the corresponding household, the probability of selection, adjustments for non-response per gender, and calibration by population totals by gender and age groups. Details on the sampling process, weighting factors, collection and other information can be found in the official publication with the PNS results^a^.

Information on access to and use of health services was obtained using data related to all household residents. Thus, PNS collected information for 205,000 residents, representing 200.6 million Brazilians^a^.

The study analyzed PNS data related to access to and use of health services according to whether users:

1.Did not perform their normal activities for health reasons in the two weeks prior to the survey;

2.Usually seek the same facility, doctor or health service when in need of health care;

3.Visited a doctor in the last 12 months;

4.Visited a dentist in the last 12 months;

5.Sought health care in the two weeks prior to the survey;

a. ... and received health care in their first visit;

b. ... and received health care;

6.Sought health care in the two weeks prior to the survey and were prescribed medication;

7.Obtained all medicines prescribed in the last visit;

8.Obtained at least one of the medicines prescribed in the last visit;

9.Obtained at least one of the medicines prescribed in the last visit from the Popular Pharmacy Program (PFP);

10.Obtained at least one of the medicines prescribed in the last visit from the public health service;

11.Were hospitalized for 24 hours or more in the 12 months prior to the survey;

a. ... by SUS, and rated the service received as good or very good;

b. ... and the last hospitalization was by SUS.

Prevalence and confidence intervals of 95% (95%CI) were estimated and stratified by age group (0–17; 18–29; 30–39; 40–59; 60 or over), level of education of head of household (no education and incomplete elementary; complete elementary and incomplete secondary; complete secondary and incomplete tertiary; complete tertiary), Brazilian macroregion (North, Northeast, Southeast, South, and Center-West) and Brazil. The differences were considered significant when no overlap between confidence intervals was identified.

Data analysis was performed with Stata software version 11.0, using the survey module, which considers effects of complex sampling. The PNS was approved by the National Research Ethics Committee (Protocol 328,159, of June 26, 2013). All interviewees were consulted, informed and accepted to participate in the research.

## RESULTS

Table 1 shows the indicators for access to and use of health services, total figures and by age group. In total, 7.0% of the population did not perform their normal activities due to health reasons in the two weeks prior to the survey, 77.8% usually sought the same doctor or health service, and 44.4% visited a dentist in the last 12 months. In addition, among the 15.3% who reported having sought the health service in the two weeks prior to the survey, 95.3% received health care in their first visit.

**Table 1:** Indicators of access to and use of health services by age group. 2013 National Health Survey.

Indicator	Total	Age Group									

		0–17		18–29		30–39		40–59		60 or over	
					
	% 95CI%	%	95CI%	%	95CI%	%	95CI%	%	95CI%	%	95CI%
Did not perform normal activities for health reasons in the two weeks prior to the survey	7.0 6.8–7.2	5.6	5.2–5.9	4.6	4.2–4.9	6.0	5.6–6.4	8.7	8.3–9.1	11.5	10.8–12.2
Usually seeks the same facility, doctor or health service when in need of health care	77.8 77.0–78.6	79.9	79.0–80.9	75.6	74.6–76.7	76.4	75.3–77.5	77.3	76.4–78.3	79.3	78.2–80.4
Visited a doctor in the last 12 months	71.2 70.7–71.7	69.8	69.0–70.6	64.1	63.1–65.1	68.4	67.4–69.4	73.5	72.8–74.3	83.5	82.8–84.2
Visited a dentist in the last 12 months	44.4 43.8–45.1	45.2	44.3–46.1	51.0	50.1–52.0	50.4	49.3–51.5	42.9	41.9–43.9	28.9	27.6–30.1
Sought health care in the two weeks prior to the survey	15.3 15.0–15.7	11.9	11.3–12.4	11.4	10.9–12.0	13.7	13.0–14.4	18.0	17.4–18.6	25.0	24.0–25.9
... and received care in the first visit	95.3 94.9–95.8	95.7	94.9–96.5	94.0	92.8–95.1	94.9	93.8–95.9	95.1	94.3–95.8	96.6	95.8–97.3
... and received care	97.0 96.6–97.4	97.6	96.9–98.2	96.3	95.4–97.2	96.5	95.5–97.4	96.6	96.0–97.3	97.8	97.2–98.4
Received health care in the last two weeks, with prescribed medicines	64.8 63.8–65.9	69.2	67.2–71.3	59.8	57.4–62.3	61.1	58.7–63.5	64.1	62.3–65.9	67.3	65.2–69.4
Obtained all medicines prescribed in the last health care visit	82.5 81.2–83.7	81.6	79.1–84.1	84.0	81.7–86.4	81.5	79.0–84.0	82.7	81.0–84.5	82.6	80.3–84.8
Obtained at least one medicine prescribed in the last health care visit	92.4 91.7–93.1	91.3	89.7–92.8	93.1	91.7–94.4	90.0	88.1–91.8	92.7	91.5–93.9	94.4	93.2–95.5
Obtained at least one medicine prescribed in the last health care visit from the Popular Pharmacy Program	21.9 20.5–23.4	16.2	13.7–18.7	16.8	14.1–19.5	14.9	12.6–17.1	25.5	23.4–27.6	30.3	27.7–32.8
Obtained at least one medicine prescribed in the last health care visit from the public health system	33.2 31.6–34.7	32.4	29.5–35.3	29.2	26.1–32.2	28.6	25.7–31.5	34.4	32.0–36.8	37.4	34.5–40.2
Was hospitalized for 24 hours or more in the 12 months prior to the survey	6.0 5.8–6.3	4.5	4.2–4.8	5.5	5.1–5.8	5.9	5.5–6.4	6.0	5.6–6.4	10.2	9.6–10.9
... by the Brazilian Unified Health System (SUS) and ranked the service as good or very good	82.4 80.9–83.9	80.5	77.6–83.5	79.7	76.4–82.9	81.3	78.0–84.6	84.2	81.7–86.6	85.5	82.7–88.2
... and the last hospitalization was by the Brazilian Unified Health System (SUS)	65.7 63.8–67.5	75.2	72.3–78.1	69.7	66.3–73.2	58.8	54.8–62.7	62.7	59.2–66.2	61.8	58.2–65.4

Regarding age groups, the study identified differences in several indicators, with the highest percentages in the group aged 60 years or over for the following indicators: not performing normal activities for health reasons in the two weeks prior to the survey (11.5%), visiting a doctor in the last 12 months (83.5%), seeking health care in the two weeks prior to the survey (25.0%), obtaining at least one of the medicines prescribed in the last visit from the PFP (30.3%) (Table 1).

The younger age groups, on the other hand, showed higher percentages than the oldest one regarding visiting a dentist in the last 12 months (51.0% for ages 18–29 and 28.9% for 60 years or over) and being hospitalized for 24 hours or more, with the last hospitalization by SUS (75.2% for ages 0–17 and 61.8% for 60 years or over). The other indicators were similar between the age groups (Table 1).

Table 2 shows the indicators of access to and use of health services according to the level of education of the head of household. Higher percentages were observed in the group of heads of household with no education to incomplete elementary for the following indicators: not performing normal activities for health reasons in the two weeks prior to the survey (7.8%), receiving health care with medicine prescription in the two weeks prior to the survey (64.8%), obtaining at least one of the prescribed medicines from the PFP (26.5%), being hospitalized for 24 hours or more, with the last hospitalization by SUS (82.5%).

**Table 2:** Indicators of access to and use of health services by level of education. 2013 National Health Survey.

Indicator	Total		Level of education of head of household							

			No education or incomplete elementary		Complete elementary or incomplete secondary		Complete secondary or incomplete tertiary		Complete tertiary	
				
	%	95CI%	%	95CI%	%	95CI%	%	95CI%	%	95CI%
Did not perform normal activities for health reasons in the two weeks prior to the survey	7.0	6.8–7.2	7.8	7.5–8.1	6.5	6.1–7.0	6.2	5.8–6.5	6.3	5.8–6.9
Usually seeks the same facility, doctor or health service when in need of health care	77.8	77.0–78.6	78.7	77.6–79.8	76.3	74.8–77.9	76.3	75.0–77.5	79.5	77.9–81.1
Visited a doctor in the last 12 months	71.2	70.7–71.7	67.1	66.4–67.9	70.8	69.6–71.9	74.3	73.5–75.2	81.0	79.9–82.2
Visited a dentist in the last 12 months	44.4	43.8–45.1	34.1	33.4–34.9	44.6	43.1–46.0	53.0	52.0–54.1	65.8	64.2–67.3
Sought health care in the two weeks prior to the survey	15.3	15.0–15.7	14.9	14.4–15.4	14.1	13.2–14.9	15.5	14.9–16.1	18.1	17.1–19.2
... and received care in the first visit	95.3	94.9–95.8	94.5	93.8–95.2	94.6	93.4–95.7	95.9	95.1–96.7	97.7	96.9–98.4
... and received care	97.0	96.6–97.4	96.4	95.7–97.0	96.4	95.5–97.4	97.5	96.8–98.2	98.8	98.3–99.4
Received health care in the last two weeks, with prescribed medicines	64.8	63.8–65.9	67.2	65.7–68.8	64.7	61.9–67.4	62.9	60.9–64.8	61.2	58.2–64.2
Obtained all medicines prescribed in the last health care visit	82.5	81.2–83.7	79.8	78.0–81.7	82.3	79.6–85.1	83.8	81.8–85.7	89.3	86.7–91.9
Obtained at least one medicine prescribed in the last health care visit	92.4	91.7–93.1	92.1	91.1–93.1	92.2	90.4–94.1	92.4	91.1–93.7	94.1	92.1–96.1
Obtained at least one medicine prescribed in the last health care visit from the Popular Pharmacy Program	21.9	20.5–23.4	26.5	24.3–28.6	21.9	18.7–25.1	19.5	17.3–21.8	10.8	8.4–13.2
Obtained at least one medicine prescribed in the last health care visit from the public health system	33.2	31.6–34.7	42.8	40.5–45.1	34.3	30.9–37.8	25.1	22.8–27.5	13.9	11.3–16.6
Was hospitalized for 24 hours or more in the 12 months prior to the survey	6.0	5.8–6.3	6.1	5.8–6.4	6.3	5.6–7.0	5.6	5.3–6.0	6.4	5.8–6.9
... by the Brazilian Unified Health System (SUS) and ranked the service as good or very good	82.4	80.9–83.9	83.3	81.6–85.1	81.6	77.4–85.9	81.0	78.1–83.9	78.1	70.7–85.6
... and the last hospitalization was by the Brazilian Unified Health System (SUS)	65.7	63.8–67.5	82.5	80.7–84.3	68.5	63.5–73.5	53.7	50.5–56.8	21.7	18.1–25.4

However, for the group of heads of household with the highest level of education, the following indicators were observed: medical and dental visits in the last 12 months (81.0% and 65.8%, respectively), seeking medical care in the two weeks prior to the survey (18.1%), receiving health care (97.7%), receiving health care in their first visit (98.8%), and obtaining all medications prescribed in the last health care visit (89.3%) (Table 2).

Table 3 shows the indicators of access to and use of health services according to the Brazilian macroregions. The South region had a higher percentage of people who did not perform their normal activities for health problems in the two weeks prior to the survey (8.4%) and of people who usually seek the same facility, doctor or health service when they need health care (83.0%).

**Table 3:** Indicators of access to and use of health services by Brazilian macroregions. 2013 National Health Survey.

Indicator	Total	Macroregion									

		North		Northeast		Southeast		South		Center-Wes	
					
	% 95CI%	%	95CI%	%	95CI%	%	95CI%	%	95CI%	%	95CI%
Did not perform normal activities for health reasons in the two weeks prior to the survey	7.0 6.8–7.2	5.8	5.4–6.3	7.8	7.5–8.2	6.2	5.8–6.6	8.4	7.8–9.0	7.0	6.5–7.5
Usually seeks the same facility, doctor or health service when in need of health care	77.8 77.0–78.6	74.2	71.9–76.4	74.3	72.7–75.8	79.6	78.3–81.0	83.0	81.2–84.9	74.8	73.1–76.6
Visited a doctor in the last 12 months	71.2 70.7–71.7	61.4	59.9–62.9	66.3	65.5–67.2	75.8	74.8–76.8	73.8	72.6–74.9	69.5	68.4–70.6
Visited a dentist in the last 12 months	44.4 43.8–45.1	34.4	33.0–35.9	37.5	36.5–38.4	48.3	47.1–49.5	51.9	50.2–53.6	44.9	43.6–46.2
Sought health care in the two weeks prior to the survey	15.3 15.0–15.7	10.1	9.4–10.7	13.4	12.8–13.9	17.1	16.4–17.8	17.9	16.9–18.9	13.8	13.1–14.4
... and received care in the first visit	95.3 94.9–95.8	93.6	92.3–95.0	93.7	92.9–94.5	96.3	95.5–97.0	96.3	95.3–97.2	93.7	92.5–95.0
... and received care	97.0 96.6–97.4	95.6	94.4–96.8	95.8	95.1–96.5	97.4	96.8–98.1	98.2	97.6–98.8	96.4	95.5–97.2
Received health care in the last two weeks, with prescribed medicines	64.8 63.8–65.9	67.1	64.5–69.8	63.5	61.8–65.3	63.8	62.0–65.6	68.5	65.9–71.0	65.5	63.3–67.7
Obtained all medicines prescribed in the last health care visit	82.5 81.2–83.7	75.8	72.8–78.8	80.8	78.9–82.6	83.1	81.0–85.3	84.8	82.2–87.5	83.0	80.7–85.2
Obtained at least one medicine prescribed in the last health care visit	92.4 91.7–93.1	91.5	90.0–92.9	90.0	88.5–91.5	92.9	91.7–94.0	94.8	93.7–96.0	92.3	90.7–93.9
Obtained at least one medicine prescribed in the last health care visit from the Popular Pharmacy Program	21.9 20.5–23.4	20.3	17.2–23.3	16.4	14.3–18.4	23.7	21.2–26.1	26.5	22.8–30.2	18.9	16.2–21.6
Obtained at least one medicine prescribed in the last health care visit from the public health system	33.2 31.6–34.7	34.8	31.7–38.0	32.4	29.7–35.1	32.4	29.8–35.0	37.2	33.8–40.6	29.1	26.2–32.1
Was hospitalized for 24 hours or more in the 12 months prior to the survey	6.0 5.8–6.3	5.8	5.4–6.3	5.6	5.3–5.8	5.7	5.3–6.1	7.5	7.0–8.0	7.4	7.0–7.9
... by the Brazilian Unified Health System (SUS) and ranked the service as good or very good	82.4 80.9–83.9	68.5	63.9–73.2	79.8	77.5–82.1	87.1	84.3–89.9	85.2	81.8–88.5	80.8	77.7–83.8
... and the last hospitalization was by the Brazilian Unified Health System (SUS)	65.7 63.8–67.5	73.9	70.9–76.9	76.5	73.9–79.1	58.8	55.0–62.7	63.8	59.3–68.2	61.6	58.3–64.9

The highest percentage of medical visits in the last 12 months was observed in the Southeast region (75.8%), and the lowest in the North region (61.4%). Differences in the percentage of dental appointments in the last 12 months were also observed. The South region showed a higher percentage (51.9%) compared to the North region (34.4%). The latter also showed the lowest percentage of people who sought health care in the two weeks prior to the survey (10.1%) compared to the other regions. Among people who reported receiving care, the figures were higher in the Southeast and South regions (97.4% and 98.2%) (Table 3).

Regarding health care with medicine prescription, the highest percentage of people who obtained all prescribed medicines was observed in the South region (84.8%) and the lowest in the North region (75.8%). In the Northeast region, 90.0% of the people obtained at least one of the prescribed medicines. The study also found differences between regions in obtaining medicines from the PFP and the public health service (Table 3).

The hospitalization percentage was higher in the South region (7.5%) compared to the others. Among people who reported hospitalization by SUS, the highest figure was in the Northeast region (76.5%). The Southeast region showed a better evaluation of care received during admission by SUS (87.1%) (Table 3).

## DISCUSSION

Access to health services in the first visit was virtually universal, despite the differences by age group, level of education, and Brazilian region. Access to medicines was also satisfactory, and 1/3 of the people who managed to obtain at least one of the prescribed medicines did so from SUS. Less than half of the Brazilian population visited a dentist in the 12 months prior to the survey, with the lowest percentages among individuals aged 60 or over; whose head of household had no education or incomplete elementary; and who resided in the North region of the Country. The findings of this study are similar those of other Brazilian studies^7,8,b^.

A study comparing the editions of the PNAD Health Supplement with PNS^8^ showed that access to and use of health services has been increasing in Brazil, although important regional differences still remain. Access is directly related to supply (availability of services geared towards the population). Difficulties in this access are, in turn, related to the peculiarities of health systems and services. Several studies suggest that access to and use of health services in Brazil reflect inequalities among different social groups^7,9,10^.

The PNS data corroborate such studies, since people living in the Southeast and South regions showed greater access to services when compared to residents of other regions, and people with higher levels of education (income proxy) showed greater access to services compared to those with lower levels of education. Other studies also found large differences in health services between Brazilian regions^11^, with higher percentages of medical visits in the South and Southeast regions, which have better living conditions and higher Human Development Indexes (HDI)^12^.

PNS assessed the reach of the Family Health Strategy in Brazil. This strategy provided better quality in primary health care, especially for the population with the lowest socioeconomic status^13^. Since the creation of SUS, services have been expanded and several activities in health services have been almost universalized, such as vaccination coverage.

Demand for health services in the two weeks prior to the survey was greater in the population with complete tertiary education. A study carried out in São Paulo reported that older adults with lower income and educational levels sought health services less frequently^14^. According to Viacava and Bellido^15^, the rates of medical appointments in the last 12 months in Brazil increased from 54.7% in 1998 to 71.2% in 2013, despite persistent differences by region and schooling. Differences in the use of services and reduced access to medical appointments by education level have also been described in a study by Barros^16^ comparing the 2003 and 2008 editions of PNAD.

The possibility of using a health service regularly can be considered an important indicator of access to the health system. In turn, access is a complex concept and relates – in addition to the offer, capacity of producing services – to the health needs perceived by the user, mediated by individual factors. Thus, such factors, as the emergence of health problems or complaints, are transformed in demands and, consequently, use of services^4,17^.

The data found in PNS show an increase compared to previous PNADs: about 78% of the Brazilian population usually seek the same facility, doctor or health service when they need health care. It is important to note that no differences were found by level of education of the head of household, indicating the importance of SUS in reducing inequalities in health.

Limitation of normal activities for health reasons is an indicator suggested by the World Health Organization for health surveys^c^, since it assesses the impact of a particular disease or problem on an individual’s daily life. As in PNS, other surveys carried out in Brazil^b^ and in the city of São Paulo^2^ have used this indicator when investigating the two-week period prior to the survey. The percentage of people who did not perform their normal activities in those two weeks was lower among those whose head of household had complete tertiary education, a pattern observed in previous PNAD editions. However, the percentage of people who did not perform their normal activities for health reasons was higher among those living in the South region, unlike the findings of the study by Travassos^7^, who found the highest percentages in the Southeast region of Brazil.

Regarding hospitalizations for 24 hours or more in the 12 months prior to the survey, higher percentages were observed in the group of heads of household with no education or incomplete elementary. The hospitalization rate was also higher in the South region, while the percentage of people whose last hospitalization was by SUS was higher in the Northeast region. This reflects the large coverage of health plans in the Southeastern and Southern regions and the low coverage in the Northeast region, expanding in the latter the participation of SUS in health care to the population^18^.

Regional inequalities were also found by Viacava et al.^19^when analyzing hospitalizations for angioplasty and revascularization surgery, used as a proxy for access to high complexity services. Brazil is a privileged setting for the debate on social inequalities, due to its long tradition of commitment to health equity. Many obstacles prevail, such as the prevailing historical inequality. The Southeast region, which has the highest HDI in Brazil, showed a lower percentage of hospitalizations by SUS, but at the same time had a better evaluation of received care.

In the area of dental visits, the World Health Organization recommends one dentist for every 1,500 inhabitants. In Brazil, there is one dentist for every 800 inhabitants. However, a low percentage of dental visits was found, especially in the population with, at most, incomplete secondary education. In addition, a difference between the Brazilian regions was identified. The highest percentages were observed in the South and Southeast regions, and the lowest in the North. The Brazil Sorridente (Smiling Brazil) program^20^, a policy for social inclusion implemented in 2006 in Brazil, has extended the population’s access to oral health, but significant inequalities persist by schooling. Oral health policy in Brazil is still expanding and seems to have contributed to the increase in the use of services in this area, but not enough to overcome the socioeconomic inequalities in the use of these services. The present study ratifies Barbato et al.^21^, who affirm: “Brazilians have in their mouths a clear picture of existing inequalities in Brazilian society as a whole” (p. 1,812).

The study identified a high percentage of access to medicines obtained from SUS or the private sector, and a high percentage of access to at least one of the medicines prescribed in the last health visit, which agrees with the literature^22^. Differences were observed between Brazilian regions regarding access to medicines in the public service and PFP (in both cases, higher in the South). Higher percentages of people who obtained medicines from the public health service or PFP were observed in the group of heads of household with no education to incomplete elementary. The results of this study show that the population with low purchasing power is supported by SUS for access to medicines and, therefore, SUS tends to equity in its attempt to universalize access to medicines^23,24^.

A limitation of this study is obtaining information from a single respondent, i.e., one person in the household answered on behalf of the other residents, potentially influencing the validity of the information^25^. Another factor was the use of level of education as income proxy, since the lack of data on household income and per capita income made it impossible to analyze access to and use of services in this perspective, which is significant in terms of health inequalities.

PNS was designed with the purpose of improving and expanding the PNAD Health Supplements, since the PNS sampling process afforded coverage of a wider geographical area and gain in estimate accuracy. To enable comparability with PNAD supplements, the blocks that investigated the use of health services were maintained, ensuring continuity of the historical series. The monitoring of indicators of access to and use of health services via population-based surveys is invaluable to assess health systems, contributing to the advancement of knowledge and improvements in health policies.

Although PNS data show improvements and advances in access to and use of health services, differences by region and level of education are still observed: the South and Southeast regions have greater access, as well as those individuals whose head of household has a higher level of education. There is great worth in aiming to reduce disparities between regions and different levels of society, since the process of implementing SUS includes the principle of universalization, guaranteed by the Constitution and regulated according to the needs of the population it serves.

The results of this study underscore the importance of monitoring the described indicators and improving policy frameworks, aiming at the better functioning of the health system.

## Footnotes

a Instituto Brasileiro de Geografia e Estatística. Acesso e utilização dos serviços de saúde, acidentes e violências. Rio de Janeiro; 2015.

b Instituto Brasileiro de Geografia e Estatística. Pesquisa Nacional por Amostra de Domicílios. Rio de Janeiro; 2009.

c World Health Organization, Regional Office for Europe. Health interview surveys: towards international harmonization of methods and instruments. Copenhagen; 1996. (WHO Regional Publications, European Series, 58).
